# Penta­aqua­[5,5′-(*m*-phenylene)ditetra­zolato-κ*N* ^2^]manganese(II) dihydrate

**DOI:** 10.1107/S1600536808028316

**Published:** 2008-09-13

**Authors:** Yuanqi Lü

**Affiliations:** a Department of Chemsitry, Dezhou University, University West Road 566, Dezhou 253023, People’s Republic of China

## Abstract

The title compound, [Mn(C_8_H_4_N_8_)_2_(H_2_O)_5_]·2H_2_O, is the fourth transition metal complex containing the 1,3-di(2*H*-tetra­zol-5-yl)benzene ligand to be structurally characterized. The Mn^II^ cation has a distorted octahedral coordination geometry. The 1,3-di(tetra­zol-5-yl)benzene ligand is planar. All H atoms bonded to O atoms participate in hydrogen bonds, which link the mol­ecules into a framework structure.

## Related literature

For similar complexes, see: Jiang *et al.* (2004 ▶); Hill *et al.* (1996 ▶).
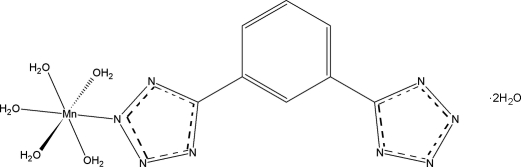


## Experimental

### 

#### Crystal data

[Mn(C_8_H_4_N_8_)_2_(H_2_O)_5_]·2H_2_O
*M* *_r_* = 393.24
Triclinic, 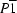

*a* = 6.5932 (1) Å
*b* = 10.0711 (2) Å
*c* = 12.9857 (3) Å
α = 68.296 (1)°
β = 77.213 (3)°
γ = 77.280 (5)°
*V* = 772.10 (3) Å^3^
*Z* = 2
Mo *K*α radiation
μ = 0.91 mm^−1^
*T* = 296 (2) K
0.26 × 0.14 × 0.08 mm

#### Data collection

Bruker SMART CCD area-detector diffractometer
Absorption correction: multi-scan (*SADABS*; Sheldrick, 1996 ▶) *T*_min_ = 0.798, *T*_max_ = 0.931
7710 measured reflections
3704 independent reflections
2846 reflections with *I* > 2σ(*I*)
*R*_int_ = 0.022

#### Refinement

*R*[*F* ^2^ > 2σ(*F* ^2^)] = 0.038
*wR*(*F* ^2^) = 0.095
*S* = 1.02
3704 reflections
273 parameters
H atoms treated by a mixture of independent and constrained refinement
Δρ_max_ = 0.36 e Å^−3^
Δρ_min_ = −0.30 e Å^−3^

### 

Data collection: *SMART* (Bruker, 2007 ▶); cell refinement: *SMART*; data reduction: *SAINT-Plus* (Bruker, 2007 ▶); program(s) used to solve structure: *SHELXS97* (Sheldrick, 2008 ▶); program(s) used to refine structure: *SHELXL97* (Sheldrick, 2008 ▶); molecular graphics: *SHELXTL* (Sheldrick, 2008 ▶); software used to prepare material for publication: *SHELXTL.*

## Supplementary Material

Crystal structure: contains datablocks global. DOI: 10.1107/S1600536808028316/ez2139sup1.cif

Structure factors: contains datablocks I. DOI: 10.1107/S1600536808028316/ez2139Isup2.hkl

Additional supplementary materials:  crystallographic information; 3D view; checkCIF report

## Figures and Tables

**Table 1:** Hydrogen-bond geometry (Å, °)

*D*—H⋯*A*	*D*—H	H⋯*A*	*D*⋯*A*	*D*—H⋯*A*
O7—H7*B*⋯O6	0.57 (6)	2.31 (6)	2.852 (3)	162 (8)
O5—H5*A*⋯O5^i^	0.57 (6)	2.41 (5)	2.910 (6)	148 (9)
O6—H6*B*⋯O7^ii^	0.61 (5)	2.21 (5)	2.814 (3)	171 (6)
O4—H4*A*⋯O1^iii^	0.67 (4)	2.38 (4)	3.035 (3)	167 (5)
O3—H3*A*⋯O7^i^	0.84 (4)	1.91 (4)	2.747 (3)	171 (3)
O3—H3*B*⋯O6^iv^	0.82 (3)	1.98 (3)	2.794 (3)	176 (3)
O2—H2*B*⋯N1^v^	0.75 (3)	2.06 (3)	2.800 (3)	173 (3)
O1—H1*B*⋯N6^vi^	0.85 (4)	1.89 (4)	2.730 (3)	176 (3)
O5—H5*B*⋯N8^vii^	0.75 (4)	2.07 (4)	2.810 (3)	168 (4)
O7—H7*A*⋯N5^ii^	0.73 (4)	2.10 (4)	2.828 (3)	173 (4)
O4—H4*B*⋯N3^iv^	0.88 (4)	1.80 (4)	2.681 (3)	175 (3)
O6—H6*A*⋯N4	0.82 (3)	2.07 (4)	2.886 (3)	176 (3)
O1—H1*A*⋯N7^viii^	0.78 (3)	1.99 (3)	2.771 (3)	175 (3)

Symmetry codes: (i) 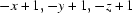; (ii) 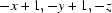; (iii) 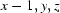; (iv) 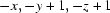; (v) 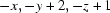; (vi) 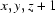; (vii) 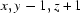; (viii) 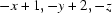.

**Fig. 1.**
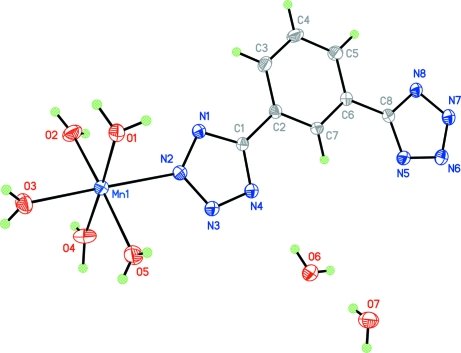
Molecular structure of (I) showing 50% probability displacement ellipsoids and the atom-labelling scheme.

**Fig. 2.**
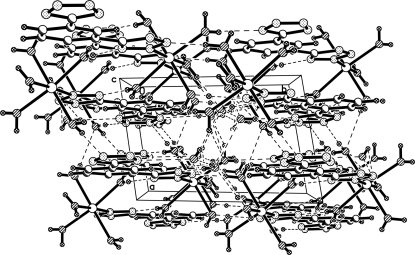
Packing diagram viewed down the c axis,

## supplementary crystallographic information

### Comment

The 1,3-di(2*H*-tetrazol-5-yl)benzene (DHTB) ligand has hitherto been reported in the twice deprotonated form in the crystal structures of its complexes with zinc, cadmium and tin (Jiang *et al.*, 2004; Hill *et al.*, 1996), where it acts as a bridging ligand. This paper provides the first structural characterization of a DHTB complex with the transition metal Mn(II); the ligand in this complex is also twice deprotonated, but coordinated to the Mn atom as a terminal ligand.

The molecule of Mn(DHTB)(H_2_O)_5_ occupies a general position in the unit cell; the Mn atom has a non-distorted octahedral coordination as indicated by bond lengths and angles (Fig. 1). The DHTB ligand has an essentially planar conformation, with the maximum deviation from the mean plane being 0.054 (2) Å by atom C7. The geometry of the ligand is similar to that observed in Jiang *et al.* (2004) and Hill *et al.* (1996).

Strong π–π interactions between the aromatic rings are indicated by the short distance of 3.324 (3) Å between C1 and C8^i^ [Symmetry code: (i) 1-x, 2-y, -z]. All hydrogen atoms that are bonded to oxygen atoms participate in H-bonding (Table 1); the extensive H-bond system and the strong π–π interactions link molecules of the complex and non-coordinated water molecules into a three-dimensional infinite network (Fig. 2).

### Experimental

The hydrothermal reaction of Mn(NO_3_)_2_ (0.5 mmol) and 1,3-di(2*H*-tetrazol-5-yl)benzene (0.5 mmol) in 20 ml of distilled water at 180°C for 3 days resulted in light yellow plate crystals of the title compound, in a yield of 42%. The crystals were filtered, washed with cold EtOH and dried in air.

### Refinement

All of the H atoms on carbon atoms were positioned geometrically and refined using a riding model with C—H = 0.93 Å and *U*_iso_(H) = 1.2 times *U*_eq_(C). All of the H atoms on oxygen atoms were located from the difference Fourier map, and refined freely, except for the bond length of O5—H5A being constrained to 0.87 Å.

### Crystal data

**Table:**

[Mn(C_8_H_4_N_8_)_2_(H_2_O)_5_]·2H_2_O	*Z* = 2
*M**_r_* = 393.24	*F*(000) = 406
Triclinic, *P*1	*D*_x_ = 1.691 Mg m^−^^3^
Hall symbol: -P 1	Mo *K*α radiation, λ = 0.71073 Å
*a* = 6.5932 (1) Å	Cell parameters from 3628 reflections
*b* = 10.0711 (2) Å	θ = 2.7–27.9°
*c* = 12.9857 (3) Å	µ = 0.91 mm^−^^1^
α = 68.296 (1)°	*T* = 296 K
β = 77.213 (3)°	Plate, yellow
γ = 77.280 (5)°	0.26 × 0.14 × 0.08 mm
*V* = 772.10 (3) Å^3^	

### Data collection

**Table:**

Bruker SMART CCD area-detector diffractometer	3704 independent reflections
Radiation source: fine-focus sealed tube	2846 reflections with *I* > 2σ(*I*)
graphite	*R*_int_ = 0.022
φ and ω scans	θ_max_ = 28.1°, θ_min_ = 1.7°
Absorption correction: multi-scan (SADABS; Sheldrick, 1996)	*h* = −8→6
*T*_min_ = 0.798, *T*_max_ = 0.931	*k* = −13→13
7710 measured reflections	*l* = −17→17

### Refinement

**Table:**

Refinement on *F*^2^	Primary atom site location: structure-invariant direct methods
Least-squares matrix: full	Secondary atom site location: difference Fourier map
*R*[*F*^2^ > 2σ(*F*^2^)] = 0.038	Hydrogen site location: inferred from neighbouring sites
*wR*(*F*^2^) = 0.095	H atoms treated by a mixture of independent and constrained refinement
*S* = 1.02	*w* = 1/[σ^2^(*F*_o_^2^) + (0.0414*P*)^2^ + 0.3671*P*] where *P* = (*F*_o_^2^ + 2*F*_c_^2^)/3
3704 reflections	(Δ/σ)_max_ = 0.001
273 parameters	Δρ_max_ = 0.36 e Å^−^^3^
0 restraints	Δρ_min_ = −0.30 e Å^−^^3^

### Special details

**Table:**

Geometry. All e.s.d.'s (except the e.s.d. in the dihedral angle between two l.s. planes) are estimated using the full covariance matrix. The cell e.s.d.'s are taken into account individually in the estimation of e.s.d.'s in distances, angles and torsion angles; correlations between e.s.d.'s in cell parameters are only used when they are defined by crystal symmetry. An approximate (isotropic) treatment of cell e.s.d.'s is used for estimating e.s.d.'s involving l.s. planes.
Refinement. Refinement of *F*^2^ against ALL reflections. The weighted *R*-factor *wR* and goodness of fit *S* are based on *F*^2^, conventional *R*-factors *R* are based on *F*, with *F* set to zero for negative *F*^2^. The threshold expression of *F*^2^ > σ(*F*^2^) is used only for calculating *R*-factors(gt) *etc*. and is not relevant to the choice of reflections for refinement. *R*-factors based on *F*^2^ are statistically about twice as large as those based on *F*, and *R*- factors based on ALL data will be even larger.

### Fractional atomic coordinates and isotropic or equivalent isotropic displacement parameters (Å^2^)

**Table:**

	*x*	*y*	*z*	*U*_iso_*/*U*_eq_	
Mn1	0.11390 (6)	0.68638 (4)	0.58413 (3)	0.02732 (12)	
O1	0.3923 (3)	0.7734 (2)	0.57901 (16)	0.0388 (4)	
O2	−0.1155 (4)	0.8808 (2)	0.58408 (19)	0.0402 (5)	
O3	0.0794 (3)	0.6043 (2)	0.76359 (15)	0.0361 (4)	
O4	−0.1679 (4)	0.6027 (2)	0.59456 (18)	0.0411 (5)	
O5	0.3064 (5)	0.4784 (2)	0.5795 (2)	0.0508 (6)	
O6	0.2643 (3)	0.5392 (2)	0.12086 (18)	0.0370 (4)	
O7	0.6605 (4)	0.3559 (3)	0.11514 (17)	0.0367 (4)	
N1	0.1613 (3)	0.90491 (19)	0.31988 (14)	0.0244 (4)	
N2	0.1633 (3)	0.76902 (19)	0.39242 (14)	0.0264 (4)	
N3	0.1980 (3)	0.6792 (2)	0.33621 (15)	0.0300 (4)	
N4	0.2200 (3)	0.75287 (19)	0.22651 (15)	0.0268 (4)	
N5	0.3286 (3)	0.9471 (2)	−0.21040 (15)	0.0276 (4)	
N6	0.3651 (3)	0.9732 (2)	−0.32077 (16)	0.0315 (4)	
N7	0.3610 (3)	1.1128 (2)	−0.37435 (16)	0.0318 (4)	
N8	0.3223 (3)	1.1812 (2)	−0.29911 (15)	0.0275 (4)	
C1	0.1974 (3)	0.8914 (2)	0.21865 (17)	0.0205 (4)	
C2	0.2128 (3)	1.0130 (2)	0.11148 (17)	0.0216 (4)	
C3	0.2001 (4)	1.1526 (2)	0.11091 (19)	0.0306 (5)	
H3	0.1796	1.1699	0.1783	0.037*	
C4	0.2177 (4)	1.2662 (2)	0.0105 (2)	0.0380 (6)	
H4	0.2077	1.3597	0.0105	0.046*	
C5	0.2501 (4)	1.2411 (2)	−0.09028 (19)	0.0311 (5)	
H5	0.2627	1.3178	−0.1576	0.037*	
C6	0.2640 (3)	1.1024 (2)	−0.09143 (17)	0.0217 (4)	
C7	0.2443 (3)	0.9883 (2)	0.00989 (17)	0.0214 (4)	
H7	0.2523	0.8950	0.0098	0.026*	
C8	0.3031 (3)	1.0766 (2)	−0.19900 (17)	0.0224 (4)	
H1A	0.455 (5)	0.808 (3)	0.519 (3)	0.044 (9)*	
H6A	0.254 (5)	0.602 (4)	0.148 (3)	0.053 (9)*	
H4B	−0.180 (5)	0.511 (4)	0.613 (3)	0.063 (10)*	
H7A	0.659 (5)	0.278 (4)	0.135 (3)	0.060 (12)*	
H5B	0.306 (6)	0.402 (4)	0.620 (3)	0.068 (12)*	
H1B	0.378 (5)	0.838 (4)	0.608 (3)	0.068 (11)*	
H2B	−0.124 (5)	0.933 (4)	0.614 (3)	0.052 (10)*	
H3B	−0.018 (5)	0.560 (3)	0.800 (3)	0.059 (10)*	
H3A	0.149 (5)	0.615 (4)	0.806 (3)	0.065 (11)*	
H4A	−0.261 (6)	0.645 (4)	0.597 (3)	0.076 (16)*	
H2A	−0.179 (7)	0.908 (5)	0.539 (4)	0.105 (18)*	
H6B	0.286 (8)	0.554 (5)	0.070 (4)	0.09 (2)*	
H7B	0.588 (10)	0.394 (6)	0.126 (5)	0.11 (3)*	
H5A	0.396 (10)	0.477 (7)	0.567 (5)	0.10 (3)*	

### Atomic displacement parameters (Å^2^)

**Table:**

	*U*^11^	*U*^22^	*U*^33^	*U*^12^	*U*^13^	*U*^23^
Mn1	0.0377 (2)	0.02288 (18)	0.02168 (18)	−0.00688 (14)	−0.00202 (14)	−0.00808 (13)
O1	0.0537 (12)	0.0406 (10)	0.0275 (9)	−0.0234 (9)	0.0075 (8)	−0.0164 (8)
O2	0.0540 (13)	0.0266 (9)	0.0423 (11)	0.0024 (8)	−0.0102 (10)	−0.0174 (9)
O3	0.0426 (11)	0.0429 (11)	0.0241 (9)	−0.0157 (9)	−0.0046 (8)	−0.0078 (8)
O4	0.0460 (13)	0.0253 (10)	0.0564 (13)	−0.0068 (9)	−0.0170 (10)	−0.0128 (9)
O5	0.0666 (16)	0.0254 (11)	0.0400 (12)	0.0045 (10)	0.0116 (11)	−0.0056 (9)
O6	0.0484 (11)	0.0342 (10)	0.0310 (10)	−0.0101 (8)	−0.0005 (9)	−0.0150 (8)
O7	0.0479 (12)	0.0276 (10)	0.0350 (10)	−0.0079 (9)	−0.0089 (8)	−0.0082 (8)
N1	0.0311 (10)	0.0217 (9)	0.0191 (8)	−0.0043 (7)	−0.0027 (7)	−0.0059 (7)
N2	0.0347 (10)	0.0215 (9)	0.0209 (9)	−0.0044 (8)	−0.0021 (8)	−0.0062 (7)
N3	0.0428 (12)	0.0239 (9)	0.0223 (9)	−0.0070 (8)	−0.0005 (8)	−0.0083 (7)
N4	0.0356 (11)	0.0240 (9)	0.0203 (9)	−0.0063 (8)	−0.0011 (8)	−0.0077 (7)
N5	0.0333 (10)	0.0258 (9)	0.0236 (9)	−0.0052 (8)	−0.0022 (8)	−0.0092 (8)
N6	0.0375 (11)	0.0343 (11)	0.0247 (10)	−0.0074 (9)	−0.0005 (8)	−0.0135 (8)
N7	0.0380 (11)	0.0333 (11)	0.0219 (9)	−0.0072 (9)	0.0009 (8)	−0.0088 (8)
N8	0.0338 (10)	0.0269 (10)	0.0193 (9)	−0.0048 (8)	0.0001 (8)	−0.0072 (7)
C1	0.0197 (10)	0.0214 (10)	0.0202 (10)	−0.0028 (8)	−0.0008 (8)	−0.0083 (8)
C2	0.0207 (10)	0.0219 (10)	0.0203 (10)	−0.0010 (8)	−0.0028 (8)	−0.0066 (8)
C3	0.0427 (14)	0.0259 (11)	0.0222 (11)	−0.0016 (10)	−0.0027 (10)	−0.0105 (9)
C4	0.0638 (18)	0.0197 (11)	0.0301 (12)	−0.0058 (11)	−0.0040 (12)	−0.0101 (9)
C5	0.0448 (14)	0.0199 (10)	0.0230 (11)	−0.0015 (10)	−0.0051 (10)	−0.0028 (9)
C6	0.0206 (10)	0.0238 (10)	0.0203 (10)	−0.0029 (8)	−0.0022 (8)	−0.0076 (8)
C7	0.0223 (10)	0.0186 (10)	0.0228 (10)	−0.0027 (8)	−0.0022 (8)	−0.0073 (8)
C8	0.0206 (10)	0.0235 (10)	0.0213 (10)	−0.0039 (8)	−0.0024 (8)	−0.0058 (8)

### Geometric parameters (Å, °)

**Table:**

Mn1—O3	2.1423 (18)	N1—N2	1.343 (2)
Mn1—O4	2.162 (2)	N2—N3	1.314 (2)
Mn1—O1	2.1797 (19)	N3—N4	1.332 (2)
Mn1—O2	2.1946 (19)	N4—C1	1.338 (3)
Mn1—O5	2.212 (2)	N5—N6	1.333 (3)
Mn1—N2	2.2857 (17)	N5—C8	1.336 (3)
O1—H1A	0.78 (3)	N6—N7	1.315 (3)
O1—H1B	0.85 (4)	N7—N8	1.343 (3)
O2—H2B	0.75 (3)	N8—C8	1.338 (3)
O2—H2A	0.73 (5)	C1—C2	1.476 (3)
O3—H3B	0.82 (3)	C2—C3	1.387 (3)
O3—H3A	0.84 (4)	C2—C7	1.394 (3)
O4—H4B	0.88 (4)	C3—C4	1.382 (3)
O4—H4A	0.67 (4)	C3—H3	0.9300
O5—H5B	0.75 (4)	C4—C5	1.385 (3)
O5—H5A	0.57 (6)	C4—H4	0.9300
O6—H6A	0.82 (3)	C5—C6	1.385 (3)
O6—H6B	0.61 (5)	C5—H5	0.9300
O7—H7A	0.73 (4)	C6—C7	1.393 (3)
O7—H7B	0.57 (6)	C6—C8	1.472 (3)
N1—C1	1.335 (3)	C7—H7	0.9300
			
O3—Mn1—O4	88.99 (8)	N3—N2—N1	109.26 (16)
O3—Mn1—O1	89.37 (8)	N3—N2—Mn1	120.96 (13)
O4—Mn1—O1	177.83 (8)	N1—N2—Mn1	129.79 (13)
O3—Mn1—O2	92.33 (8)	N2—N3—N4	109.71 (17)
O4—Mn1—O2	81.61 (9)	N3—N4—C1	104.97 (17)
O1—Mn1—O2	97.05 (8)	N6—N5—C8	105.15 (17)
O3—Mn1—O5	89.42 (8)	N7—N6—N5	109.76 (17)
O4—Mn1—O5	90.03 (11)	N6—N7—N8	109.04 (17)
O1—Mn1—O5	91.36 (10)	C8—N8—N7	105.04 (18)
O2—Mn1—O5	171.42 (11)	N1—C1—N4	111.28 (17)
O3—Mn1—N2	177.82 (7)	N1—C1—C2	124.65 (18)
O4—Mn1—N2	92.24 (8)	N4—C1—C2	124.07 (18)
O1—Mn1—N2	89.44 (7)	C3—C2—C7	119.41 (19)
O2—Mn1—N2	89.63 (8)	C3—C2—C1	120.24 (19)
O5—Mn1—N2	88.78 (8)	C7—C2—C1	120.34 (18)
Mn1—O1—H1A	116 (2)	C4—C3—C2	120.2 (2)
Mn1—O1—H1B	118 (2)	C4—C3—H3	119.9
H1A—O1—H1B	102 (3)	C2—C3—H3	119.9
Mn1—O2—H2B	131 (3)	C3—C4—C5	120.2 (2)
Mn1—O2—H2A	115 (4)	C3—C4—H4	119.9
H2B—O2—H2A	113 (4)	C5—C4—H4	119.9
Mn1—O3—H3B	120 (2)	C6—C5—C4	120.4 (2)
Mn1—O3—H3A	129 (2)	C6—C5—H5	119.8
H3B—O3—H3A	110 (3)	C4—C5—H5	119.8
Mn1—O4—H4B	127 (2)	C5—C6—C7	119.31 (19)
Mn1—O4—H4A	119 (4)	C5—C6—C8	119.95 (19)
H4B—O4—H4A	113 (4)	C7—C6—C8	120.73 (18)
Mn1—O5—H5B	132 (3)	C6—C7—C2	120.44 (19)
Mn1—O5—H5A	117 (7)	C6—C7—H7	119.8
H5B—O5—H5A	99 (7)	C2—C7—H7	119.8
H6A—O6—H6B	119 (5)	N5—C8—N8	111.01 (18)
H7A—O7—H7B	120 (6)	N5—C8—C6	125.20 (18)
C1—N1—N2	104.79 (16)	N8—C8—C6	123.78 (19)
			
C1—N1—N2—N3	−0.3 (2)	N4—C1—C2—C3	−176.0 (2)
C1—N1—N2—Mn1	−179.63 (15)	N1—C1—C2—C7	−177.9 (2)
O4—Mn1—N2—N3	−62.82 (18)	N4—C1—C2—C7	2.7 (3)
O1—Mn1—N2—N3	118.54 (17)	C7—C2—C3—C4	0.2 (4)
O2—Mn1—N2—N3	−144.41 (18)	C1—C2—C3—C4	179.0 (2)
O5—Mn1—N2—N3	27.16 (18)	C2—C3—C4—C5	−0.6 (4)
O3—Mn1—N2—N1	−119.2 (19)	C3—C4—C5—C6	0.4 (4)
O4—Mn1—N2—N1	116.41 (19)	C4—C5—C6—C7	0.2 (4)
O1—Mn1—N2—N1	−62.23 (19)	C4—C5—C6—C8	−178.7 (2)
O2—Mn1—N2—N1	34.82 (19)	C5—C6—C7—C2	−0.5 (3)
O5—Mn1—N2—N1	−153.6 (2)	C8—C6—C7—C2	178.31 (19)
N1—N2—N3—N4	0.2 (2)	C3—C2—C7—C6	0.3 (3)
Mn1—N2—N3—N4	179.52 (14)	C1—C2—C7—C6	−178.46 (19)
N2—N3—N4—C1	0.1 (2)	N6—N5—C8—N8	0.2 (2)
C8—N5—N6—N7	−0.3 (2)	N6—N5—C8—C6	−178.4 (2)
N5—N6—N7—N8	0.4 (3)	N7—N8—C8—N5	0.0 (2)
N6—N7—N8—C8	−0.2 (2)	N7—N8—C8—C6	178.67 (19)
N2—N1—C1—N4	0.4 (2)	C5—C6—C8—N5	176.9 (2)
N2—N1—C1—C2	−179.00 (19)	C7—C6—C8—N5	−1.9 (3)
N3—N4—C1—N1	−0.3 (2)	C5—C6—C8—N8	−1.6 (3)
N3—N4—C1—C2	179.09 (19)	C7—C6—C8—N8	179.6 (2)
N1—C1—C2—C3	3.3 (3)		

### Hydrogen-bond geometry (Å, °)

**Table:**

*D*—H···*A*	*D*—H	H···*A*	*D*···*A*	*D*—H···*A*
O7—H7B···O6	0.57 (6)	2.31 (6)	2.852 (3)	162 (8)
O5—H5A···O5^i^	0.57 (6)	2.41 (5)	2.910 (6)	148 (9)
O6—H6B···O7^ii^	0.61 (5)	2.21 (5)	2.814 (3)	171 (6)
O4—H4A···O1^iii^	0.67 (4)	2.38 (4)	3.035 (3)	167 (5)
O3—H3A···O7^i^	0.84 (4)	1.91 (4)	2.747 (3)	171 (3)
O3—H3B···O6^iv^	0.82 (3)	1.98 (3)	2.794 (3)	176 (3)
O2—H2B···N1^v^	0.75 (3)	2.06 (3)	2.800 (3)	173 (3)
O1—H1B···N6^vi^	0.85 (4)	1.89 (4)	2.730 (3)	176 (3)
O5—H5B···N8^vii^	0.75 (4)	2.07 (4)	2.810 (3)	168 (4)
O7—H7A···N5^ii^	0.73 (4)	2.10 (4)	2.828 (3)	173 (4)
O4—H4B···N3^iv^	0.88 (4)	1.80 (4)	2.681 (3)	175 (3)
O6—H6A···N4	0.82 (3)	2.07 (4)	2.886 (3)	176 (3)
O1—H1A···N7^viii^	0.78 (3)	1.99 (3)	2.771 (3)	175 (3)

Symmetry codes: (i) −*x*+1, −*y*+1, −*z*+1; (ii) −*x*+1, −*y*+1, −*z*; (iii) *x*−1, *y*, *z*; (iv) −*x*, −*y*+1, −*z*+1; (v) −*x*, −*y*+2, −*z*+1; (vi) *x*, *y*, *z*+1; (vii) *x*, *y*−1, *z*+1; (viii) −*x*+1, −*y*+2, −*z*.
